# Use of Narrow-Diameter Implants in Completely Edentulous Patients as a Prosthetic Option: A Systematic Review of the Literature

**DOI:** 10.1155/2021/5571793

**Published:** 2021-06-22

**Authors:** S. Storelli, A. Caputo, G. Palandrani, M. Peditto, M. Del Fabbro, E. Romeo, G. Oteri

**Affiliations:** ^1^Department of Biomedical, Surgical and Dental Sciences, University of Milan, Italy; ^2^Department of Biomedical and Dental Sciences and Morphofunctional Imaging, University of Messina, Italy

## Abstract

**Objective:**

The objective of the present review is to assess the implant survival, marginal bone loss, and biomechanical features of narrow-diameter implants (2.5-3.5 mm) supporting or retaining full-arch fixed or removable restorations.

**Materials and Methods:**

Three operators screened the literature (PubMed, Cochrane Library, and Google Scholar) and performed a hand search on the main journals that focus on implantology until 24 March 2019. Only articles that considered full-arch restorations supported or retained by narrow-diameter implants (2.5-3.5 mm) were considered if they have a minimum of 10 patients and a mean follow-up of at least 6 months. The outcome variables were survival of implants and marginal bone loss. The review was performed according to the PRISMA statements. Risk of bias assessment was evaluated. Failure rates were analyzed using random effect Poisson regression models to obtain the summary estimate of 5-year survival rate and marginal bone loss.

**Results:**

A total of nine papers were finally selected, reporting a high survival rate of the implants. Eight studies focused only on the mandible while one study reported data from both mandible and maxilla. All studies reported on removable restorations; none focused on fixed rehabilitations. The estimated survival rate for 5 years of follow-up was calculated to be 92.25% for the implants. The estimated marginal bone loss after 5 years was calculated to be 1.40 mm. No study reported implant fractures.

**Conclusions:**

With the limitations of the present study, there is evidence that 2.5-3.5 mm narrow-diameter implants retaining a removable restoration can be a successful treatment in fully edentulous patients. No data on fixed restorations was available.

## 1. Introduction

Complete edentulism significantly reduces masticatory function, and it is a significant oral health issue concerning a large part of the adult population [1].

Following tooth extraction, a process of residual ridge resorption (RRR) begins, and it is most intense during the first year, when approximately 60% of the alveolar ridge is resorbed and is directly related to the duration of edentulism [2–4].

Human reentry studies showed horizontal bone loss of 29-63% and vertical bone loss of 11-22% after 6 months following tooth extraction [5].

However, the bone resorption activity continues slowly throughout life, resulting in the loss of a large amount of jawbone structure [6].

Residual ridge resorption (RRR) is a common problem, and it represents a chronic, progressive, irreversible, and disabling disease, probably of multifactorial origin [6].

Most resorption occurs in the alveolar process, whereas the basal portion remains relatively intact but only decreases bone density due to reduced function [6].

The edentulous arch is a vital structure present during the entire life of the patient, regardless of tooth presence or function [7]. All this often leads to a situation where there is not sufficient support for the appropriate function of the removable complete denture.

Conventional dentures represent a noninvasive option for the treatment of complete edentulism and avoid a surgical procedure for the patient. However, in a large number of cases, this rehabilitation does not satisfy the patients' expectations, as many complaints are reported primarily related to functionality and adaptation [8].

On the contrary, the attachment of removable dentures to osseointegrated dental implants brings considerable benefits, including the increase of denture stability, functional efficiency, and comfort. For these reasons, the implant-supported overdenture has become a common clinical practice, and to date, a two-implant overdenture is considered the first choice for the treatment of the fully edentulous mandible [9, 10].

However, in some cases, the inevitable resorption of the alveolar ridges after tooth extraction can make the placement of standard-diameter implants (>3.5 mm) difficult or even impossible without the use of more advanced bone regenerative procedures.

The use of standard-diameter implants in narrow alveolar ridges may lead to thin buccal or lingual bone or even large dehiscences, increasing the risk of complication and failure [11, 12].

In addition, elderly patients and, especially, patients with comorbidities are often unwilling to undergo extensive surgical procedures such as bone regeneration in order to receive standard-diameter implants [13]. Adapting dimensions of dental implants allows simpler surgical procedures.

In many cases, the interforaminal height can be reduced to <10 mm (Class D or E according to Lekholm and Zarb or Class IV according to the classification system for edentulous patients of the American College of Prosthodontics) [14, 15].

This situation represents the most complex to rehabilitate.

NDIs (narrow-diameter implants) 10 mm long may be too long for them, while short, wide implants may be too wide for thin residual ridges [16].

Such patients represent the most complex and high-risk treatment situation. MDIs 10 mm long may be too long for them, whereas short and wide implants may be too wide for slim residual ridges [17, 18].

To date, there are few studies in the literature on the use of short implants for the rehabilitation of edentulous arches.

In the case of a narrow ridge, two options are available. The first option is to place a standard-diameter implant secondly to bone augmentation procedures [11]; the second option is to use a narrow-diameter implant (NDI < 3.5 mm) [11].

Although widely validated in literature, bone augmentation procedures are much more invasive and may have a higher risk of complications than conventional placement of implants.

Narrow-diameter implants (NDIs) have been introduced as an alternative treatment option in single-tooth gaps or edentulous ridges with limited width [19, 20].

The reduced-diameter implant classification was updated in 2018 (Table 1) [21].

The advantages of NDIs include the following: (1) no bone grafting, (2) reduced bleeding, (3) minimal postoperative discomfort, (4) lower costs for the patients, and (5) faster healing time [21, 22].

However, NDIs also present some disadvantages: (1) reduced bone-to-implant contact (BIC) and osseointegration [20], (2) increased risk of implant fracture due to reduced mechanical properties, and (3) increased risk of implant overloading.

However, it is recommended that NDIs are used with caution. The reduction in implant diameter reduces the contact surface between bone and implant, thus increasing the risk of fracture due to reduced mechanical stability [23].

Many publications demonstrate the risk of “fatigue” fracture of small-diameter implants [20, 24].

The neck of the implant represents a potential fracture zone when subjected to high bending force. Due to these mechanical limitations, the NDIs are only recommended to increase retention and stability of mandibular overdenture in cases of limited bone thickness [25, 26].

To reduce this risk, new alloys have been introduced.

Preliminary results obtained in one study showed that reduced-diameter implants with a titanium-zirconium alloy can withstand masticatory forces in total rehabilitations [11].

The present review has the primary objective to evaluate the survival rate, the marginal bone loss (MBL), and biomechanical features of NDIs of 2.5 to 3.5 mm of diameter (categories 2 and 3) [21] used in the treatment of completely edentulous patients through full-arch removable or fixed restorations.

## 2. Materials and Methods

This systematic review was written according to the guidelines indicated by the “PRISMA statement” and by the *Cochrane Handbook for Systematic Reviews of Interventions* (version 5.1.0).

The focused question was “What is the survival rate of narrow-diameter implants (2.5-3.5mm) supporting removable or fixed restorations in fully edentulous patients?” A preliminary PICOS assessment was used to define the search strategy with the following criteria.

### 2.1. Participants

The participants are edentulous patients (both jaws or either upper or lower jaw) with a full-arch implant-retained fixed or removable prosthesis.

### 2.2. Interventions

The interventions are full-arch fixed or removable overdenture prosthesis supported by narrow-diameter implants (2.5 to 3.5 mm).

### 2.3. Outcome Measures

- Implant survival rate
- Prosthesis survival rate
- Marginal bone loss

Other variables were searched and described when present: biomechanical features, prosthesis survival, prosthetic complications, reconstruction material, and implant system used.

### 2.4. Types of Studies

The types of studies are randomized controlled trials (RCTs) and prospective and retrospective clinical trials (case-control studies, cohort studies, and case series). Studies had to report data on a minimum of 10 participants and have a minimum of 6 months of follow-up.

### 2.5. Search Strategy

**Figure 1:**
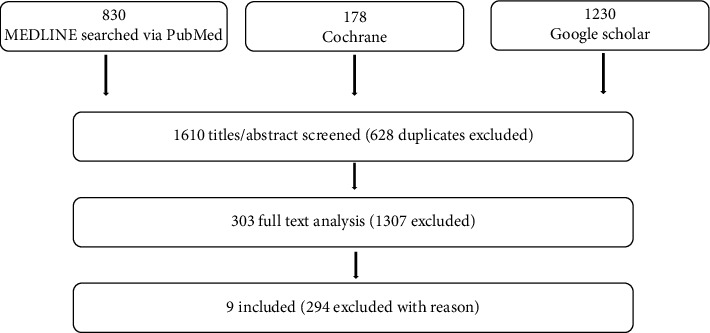


Three investigators conducted an independent electronic search of the English literature (AC, GP, and SS), using PubMed, Cochrane Library, and Google Scholar for studies published until March 2019, including the following search strategy (MeSH and free terms) for each database (Figure 1):

- (edentulous) AND ((((((((small diameter implant) OR small-diameter implant) OR narrow implant) OR mini-implant) OR mini implant) OR transitional implant) OR temporary implant) OR provisional implant): 830 hits
- (Edentulous) AND (small diameter implant OR small-diameter implant OR narrow implant OR mini-implant OR mini implant OR transitional implant OR temporary implant OR provisional implant)”: 178 hits
- (“edentulous”) AND (“small diameter implant” OR small-diameter implant OR narrow implant OR mini-implant OR “mini implant” OR transitional implant OR temporary implant OR provisional implant): 1230 hits

Moreover, the issues from January 2016 to March 2019 of the following journals were hand-searched: *Clinical Oral Implants Research*, *International Journal of Periodontics and Restorative Dentistry*, *Journal of Periodontology*, *Journal of Clinical Periodontology*, *International Journal of Oral and Maxillofacial Implants*, *Journal of Prosthetic Dentistry*, *Journal of Prosthodontics*, and *Journal of Oral Rehabilitations*.

Moreover, the bibliographies of previous systematic reviews on the topic as well as selected articles were thoroughly screened.

### 2.6. Inclusion Criteria

- Studies published in English
- Human studies
- Sample size ≥ 10 patients
- Studies conducted on completely edentulous patients rehabilitated with small-diameter implants (2.5-3.5 mm)
- Full-arch removable or fixed full-arch restorations
- Follow-up times greater than six months
- Prospective cohort studies, randomized controlled or nonrandomized controlled trial, retrospective case-control or “single cohort” studies, and case series

### 2.7. Exclusion Criteria

- Studies reporting data of the same cohort of patients, with different follow-ups
- Case reports on animal or in vitro models

### 2.8. Study Selection

All retrieved articles were screened for duplicates by two reviewers (GP-AC). Titles and abstracts were then independently screened by the same review authors (GP-AC). Articles meeting the inclusion criteria and those whose abstract presented unclear data were collected as full text. The papers were then assessed by three authors (GP-AC-MP) that defined if the articles were to be included or not. Any disagreement was resolved by discussion with the other reviewers (SS-GO).

### 2.9. Data Extraction

Data were extracted by three reviewers (AC-GP-MP) using data collection forms [27].

Study design, implant manufacturer, and data on restorations were extracted. The survival rates of implant and prosthesis were extracted. Implant survival was considered if the implant was present at the follow-up examination. Moreover, when reported, data on marginal bone loss and patient satisfaction was also extracted. When the reported data were unclear, authors contacted by emails the corresponding authors and asked for more information.

### 2.10. Risk of Bias Assessment

The risk of bias assessment for the included studies was performed independently by two reviewers (GP and AC) using The Cochrane Collaboration's tool for assessing the risk of bias including the following domains: allocation concealment, random sequence generation, blinding of participants and personnel, blinding of outcome assessment, incomplete outcome data, selective reporting, and other bias.

The assessment was not centered on the outcomes of the paper but the ones of the present review.

Each domain was considered at low, unclear, or high risk of bias agreeing to the evaluation criteria as reported in the *Cochrane Handbook for Systematic Reviews of Interventions* version 5.1.0.

After judgment was given for each of the domains mentioned above, studies were divided into the following groups: studies with low risk of bias, if all domains were considered at low risk of bias; unclear risk of bias, if one or more domains were considered at unclear risk of bias; and high risk of bias, if one or more domains were reported at high risk of bias. Cases of disagreement were resolved by discussion.

### 2.11. Statistical Analysis

The mean follow-up duration was directly extracted by the articles, provided by adjunctive information by the authors or estimated from the original data. For further analysis, the total number of events was considered to be Poisson distributed for a given sum of implant exposure years, and five-year survival and success rates for implants and prosthesis were estimated considering a Poisson distribution [27, 28]. Data were tabulated and analyzed using the software Microsoft Excel 2016 (©2016 Microsoft Corporation, Santa Rosa, CA, USA) and the software GraphPad Prism 5.0 (GraphPad, San Diego, CA, USA). For the data synthesis, weighted mean values, median, 95% confidence intervals, and ranges were used. Distribution of implant failures was assessed using a time-to-event analysis. Studies that did not provide information regarding the timing of implant loss were excluded from the analysis. Life table analysis and Kaplan-Meier analysis were used to estimate the cumulative implant survival rate. The cumulative survival rates of implants with diameter 2.5 to 3.3 mm and 3.3 to 3.5 mm were compared using the log-rank (Mantel-Cox) test. The significance threshold was set at *p* = 0.05. For each study, the estimated MBL at five years was calculated by dividing the MBL reported by years of follow-up and multiplying for five, assuming a constant marginal bone loss over time [27]. All analyses were performed using IBM SPSS Statistics (IBM Corporation, Armonk, NY).

## 3. Results

The electronic search identified a total of 2238 (830 MEDLINE, 178 Cochrane database, and 1230 Google Scholar) (Table 2).

The manual research did not produce any additional article. After removing duplicate papers, a total of 1610 were screened. A total of 303 papers underwent full-text analysis. After full-text reading, 294 papers were excluded. Reasons for exclusion are listed in Table 3.

Any disagreement was resolved by discussion. When, after discussion, there was still doubt, authors were contacted by email and asked for better explanations. Finally, nine papers were included. Therefore, nine studies were actually selected and included in the review (Figure 1).

### 3.1. Excluded Studies

Each full text was read, and the reason for exclusion is reported in Table 3. Out of 294 excluded papers, 183 reported on implants with diameter > 3.5 mm or <2.5 mm, 55 had a pool of implants with mixed diameter, 9 reported nonhuman studies, 11 reported on rehabilitations with bone regenerations, 21 analyzed partially edentulous patients, 6 reported data with follow-up less than 6 months, 3 reported data on the same pool of patients (a third one was included), 1 was a review, 4 were studies in languages other than English, and 1 reported a pool of patients less than 10.

### 3.2. Study Characteristics

Finally, a total of 9 papers [29–37] were included in the qualitative analysis. Descriptive data regarding the characteristics of included studies are reported in Table 4. Four RCTs and 4 prospective studies and 1 retrospective study were selected (Table 4).

### 3.3. Risk of Bias

The risk of bias summary is presented in Table 5. Among the studies, 4 were classified as high risk of bias [29, 34, 36, 37] and 5 were classified as unclear risk of bias [30–33, 35].

### 3.4. Data Analysis

The included studies were four randomized controlled trials [30, 32, 33, 35], four prospective studies [29, 31, 34, 37], and 1 retrospective study [36]. All studies reported cases of fully edentulous patients and implant placement with narrow-diameter implants (*Ø* 2.5-3.5 mm). Eight studies investigated only the edentulous mandible and 1 investigated both maxilla and mandible (Table 4). A total of 885 NDIs (*Ø* 2.5-3.5 mm) in 398 patients were followed for at least six months (range 6-91). Implant diameter, implant length, and implant system are shown in Table 4. All included studies reported data on removable overdentures. No data was found on fixed restorations supported by NDI in completed edentulous patients. Five studies used flap reflection techniques for implant placement [31–33, 36, 37], three studies used a flapless technique [29, 30, 34], and, in the remaining study, the technique was not specified [34] (Table 4). All the studies reported the implant survival rate with a range from 83.3% to 100%; only three studies reported the implant success rate with a range from 51.3% to 94%. The estimated survival rate after five years (%) derived from Poisson regression analysis varied between 81.7% and 100% (Table 6).

After normalization of the results, the weighted mean was 96.75% with a median of 98.35% and a 95% CI of 90.87% to 100%. The life table analysis showed results of up to 10 years of follow-up for a minimum number of implants, with a cumulative 10 years of follow-up of 95.85 (Table 7).

**Figure 2:**
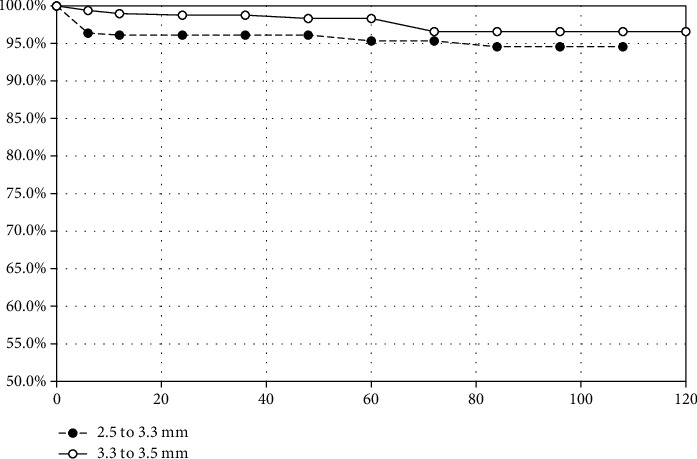
Kaplan-Meier analysis divided by implant diameter range.

The Kaplan-Meier analysis was divided by diameter range into two groups 2.5-3.25 mm and 3.3-3.5 mm. The curves have been compared using the log-rank (Mantel-Cox) test. There is a significant difference (*p* = 0.01), due to a greater number of early events (implant losses) in the group of implants with 2.5 to 3.25 mm diameter. The main contribution comes from the study by Marcello-Machado et al., in which there were 10 failures (out of 17 in this group) in the first year (4 within 2 months, 1 at 3 m, 3 at 4 m, 1 at 5 m, and 1 at 7 m) (Figure 2).

One implant failed in each test group in two articles [29, 31], six implants in Morneburg's study, and three implants in Muller's due to peri-implant infection. Ma et al. reported that 17 implants failed without explaining the reasons for failure. Marcello-Machado reported 10 implants lost out of 60 in the first year, thus leading to a very high failure rate over the first year. No one-piece to two-piece implant fractures occurred in the reviewed articles. All nine studies reported the bone level changes at the end of each follow-up from 6 months to 7.6 years. The MBL (marginal bone loss) reported in the nine studies ranged from 0 mm to 2.82 mm, and the mean MBL estimated after five years was 1.40 mm with a median of 1.24 mm and a 95% CI of 0.55 to 2.03 mm (Table 6). All the papers reported implant loading time: four studies followed conventional loading, after 3 months from implant placement [31, 33, 34, 37]; three studies followed early loading protocols, from 1 to 3 months from implant placement [32, 35, 36]; and one article followed immediate loading, within 7 days from implant placement [30]. Only one article followed both immediate and conventional loading [29] (Table 4). About the prosthetic rehabilitation, all patients were restored with removable overdentures. In all the studies, either a ball or a locator attachment was used; only in one study was a bar considered [33]. Seven studies described mandibular overdentures supported either by two implants [29–32, 34–37], by four implants, or by three implants [31], while only one of the included studies evaluated maxillary overdentures supported by three or four implants [33] (Table 4). The interforaminal area was preferred for implant placement in the mandible. In the maxilla, implant positions were not described. A prosthesis was considered to have survived whenever, although some modification during the observation time was made, the rehabilitation was still in situ at the end of the observation period [27]. Only four studies reported data about prosthesis survival: three studies reported survival of 100% after six months, two years, and three years, respectively [31, 32, 36] and one of 85% after one year [30]. The most frequent prosthetic complications were healing abutment loosening, loosening of the locator/ball attachment, and replacement of retentive cap. The fracture of mandibular overdentures was found in six cases at one year [29] and twenty-seven cases at ten years [33]. Only two studies assessed the patient's satisfaction with the overdenture by validated questionnaires based on a visual analog scale (VAS) at one year and six months, respectively [30, 32]. In these questionnaires, patients indicate their satisfaction with a crossed mark on a scale from 0 to 100 (from not at all satisfied to extremely satisfied). In both studies, the overall patient satisfaction with the overdenture was high (score > 60). The ability to speak, the level of comfort, the stability of the dentures, perception of the chewing ability, and function showed similar improvement in both studies pre- and postimplant placement. In particular, in Aunmeungton's study, the average patient satisfaction in Groups 1 (two narrow implants Ø 3 mm), 2 (four narrow implant Ø 3 mm), and 3 (standard implant Ø 3.5 mm) was 67.83 ± 5.26, 70.88 ± 4.12, and 60.85 ± 8.54, respectively. There were no significant differences in patient satisfaction between Groups 1 and 2. However, patient satisfaction in these two groups was statistically higher than Group 3.

## 4. Discussion

Up to date, only a few comparative prospective clinical studies are available to document survival or success rates of NDI. Therefore, the authors decided to also include observational studies in this review.

The quality and level of evidence were limited in general with a high risk of bias, so the interpretation of this data requires caution.

It is interesting to note that all included studies were describing overdenture rehabilitations while no studies were found concerning full-arch restorations supported by NDI.

Only two papers compared narrow with standard implants. Augmentonn compared three groups, in Groups 1 and 2, two and four minidental implants, respectively, were placed and immediately loaded by overdentures, using Equator^VR^ attachments. In Group 3, conventional implants were placed. After osseointegration, the implants were loaded by overdentures, using ball attachments.

There was no significant difference (*p* < 0.05) in clinical results regarding the number (two or four) of minidental implants with Equator attachments. However, there was a significant difference in marginal bone loss and patient satisfaction between those receiving minidental implants with Equator attachments and conventional dental implants with ball attachments.

In the Jawad study, forty-six patients were randomly allocated to receive either two mini-implants or two conventional implants in the mandible to retain their lower dentures, and no statistical differences were found.

The majority of investigated studies reported narrow implant survival rates > 95%, and only one study reported survival rates of 83.3% after 1 year. The weighted average of the estimated survival rate at five years, for removable restorations, was 96.75%.

The highest incidence of failure was found in the Marcello-Machado et al. study [37]. A total of 30 patients with 60 implants suffered from 10 implant losses in 10 different patients during the healing phase. The lost implants (2.9 mm in diameter) were replaced by a 3.5 mm diameter implant. The short follow-up of the study is one of the limits of the paper, and the 5 years of estimated survival leads to an 81.7% survival. Another study with a high failure rate was the only study describing upper jaw rehabilitations [33]. The cumulative survival rate was calculated to be 84.7% at ten years. This finding seems different if compared to those reported in the literature for standard-diameter implants. A study conducted by Fisher showed an implant survival rate of 95% at five years in edentulous patients rehabilitated with upper fixed prosthesis supported by 5-6 standard-diameter implants. As shown in Kern's review, the estimated 5-year survival rates of regular-diameter implants were 97.9% (95% CI 97.4; 98.4) in the maxilla and 98.9% (95% CI 98.7; 99.1) in the mandible. The reliability of the use of NDI in completely edentulous patients is given also by the fact that there are studies with 10 years of follow-up, although with a limited number of implants.

One of the issues of the use of NDI is the fracture rate. Although described in literature, no included studies have reported such an occurrence.

Implant diameter is related to the risk of implant fracture, with reduced diameter associated with reduced mechanical stability and increased risk of overload [31].

Narrow-diameter implants have been suggested to have less resistance to mechanical forces, when compared to standard-diameter fixtures, and may increase stress transmitted to the bone.

On the other hand, El-Sheikh et al. [31] and Morneburg [34] reported no implant fractures during 6 years and 2 years of follow-ups, respectively.

According to Morneburg's study, implant fracture was avoided thanks to proper loading protocol, placement of implants in the anterior mandible, and the use of short attachments [34].

Another issue is the prosthodontic success and complication rate. Ma et al. reported the 10-year complication rate to be very high, with only 35% of patients not needing any adjustment over time. By contrast, the patients treated in Ma et al.'s study were rehabilitated with acrylic resin without any metal framework reinforcement showing a very low number of overdenture fractures [33].

Although the data show a high survival rate, it is important to consider also the implant's success rate, health of soft tissues, and changes in the marginal bone level. Only three studies reported the implant success rate with a range from 51.3% to 94%. Studies did not present well-defined success criteria. Because of limited data, it was not possible to analyze the success rate.

The average MBL value after five years was 1.40 mm similar to those reported for standard implants in several articles. Müller et al. [35] measured a bone level change of 0.60 mm confirming the favorable results of Ti-Zr ND implants. Romeo et al. compared Straumann tissue-level implants with a reduced diameter (3.3 mm) with standard-diameter implants (4.1 mm) in partially edentulous patients and found no statistically significant difference in the MBL [12]. Moreover, there was only one study comparing narrow-diameter implants with standard dental implants (Aunmeungtong) for complete dentures, proving that the marginal bone resorption in standard implants was significantly higher than in narrow implants.

Several studies compared restorations with different numbers of narrow implants. All studies have reported the use of 2 NDI retaining a mandibular overdenture. Two studies have also included the use of 3 or 4 implants. All studies have reported the use of unsplinted implants while one reported also about a bar on 4 implants. Ball attachments are related to a decreased marginal bone loss after 3 years of follow-up regardless of implant diameter, due to a better stress distribution under peri-implant conditions when compared to the rigid connection of locator attachments.

El-Sheikh et al. [31] concluded that there were no significant differences among the considered clinical or radiographic parameters of the peri-implant tissues between two-implant overdenture versus three-implant overdenture.

As shown by Aunmeungtong et al. [30], two NDIs can be used for mandibular overdentures without any significant difference regarding marginal bone level changes and prosthodontic complications when compared to four-NDI-retained overdentures.

No study was reported on fixed restoration supported by NDI.

Only two studies assessed patients' satisfaction with the overdenture by validated questionnaires based on a visual analog scale (VAS) at one year and 6 months, respectively [30, 32].

In both studies, the overall patient satisfaction with the overdenture was high (score > 60). The results of the questionnaire in Morneburg's study confirm that stabilization of mandibular dentures with reduced-diameter implants leads to considerable improvement of the function of the prosthesis and increased comfort for the patient.

Clinical data, from this systematic review, suggest that NDIs of 2.5 mm to 3.3 mm of diameter represent an alternative treatment option in the rehabilitation of a completely edentulous jaw with limited width. This could be considered a possible alternative to bone augmentation, when needed. In a recent study by Papadimitriou et al., the rehabilitation of edentulous patients using NDIs showed to require significantly less bone regeneration. At the moment, there are no studies that compare narrow-diameter implant versus standard diameter with bone augmentation procedures in fully edentulous patients.

## 5. Conclusions

In conclusion, narrow-diameter dental implants show high survival (>95%) and acceptable, marginal bone level changes (<1.5 mm). Although the selected papers are difficult to compare, the results consider NDI a reliable treatment when used to retain an overdenture in the rehabilitation of edentulous jaw with survival rates and biomechanical features similar to standard-diameter implants.

It is possible to consider two-/three-implant overdentures in the mandible as a viable treatment option for edentulous patients with high survival rates.

No studies were found concerning rehabilitation fixed restorations in both mandible and maxilla. Only one study reported edentulous maxilla restored with overdentures supported by NDI.

Future RCTs should investigate the rehabilitation of edentulous patients with narrow implants vs. standard-diameter implants, as well as fixed restorations for edentulous patients.

## Figures and Tables

**Table 1:** Reduced-diameter implant classification.

Category	Diameter
1	<2.5 (mini-implants)
2	2.5 mm to <3.3 mm
3	3 mm to 3.5 mm

**Table 2:** Search strategy used and hits for each searched database.

Database of published studies	Search strategy used	Hits
MEDLINE searched via PubMed searched on March 24, 2019	(edentulous) AND ((((((((small diameter implant) OR small-diameter implant) OR narrow implant) OR mini-implant) OR mini implant) OR transitional implant) OR temporary implant) OR provisional implant)	830
Cochrane Central Register of Controlled Trials searched on March 20, 2019	(edentulous) AND (small diameter implant OR small-diameter implant OR narrow implant OR mini-implant OR mini implant OR transitional implant OR temporary implant OR provisional implant)	178
Google Scholar searched on March 23, 2019, via http://www.scholar.google.com/	(“edentulous”) AND (“small diameter implant” OR small-diameter implant OR narrow implant OR mini-implant OR “mini implant” OR transitional implant OR temporary implant OR provisional implant)	1230
		TOT 2238

**Table 3:** Excluded papers with reasons.

Reason of exclusion	Number of excluded studies
Mean follow‐up < 6 months	6
Mixed diameter	55
Partially edentulous	21
Out of topic (diameter > 3.5 mm or <2.5 mm)	183
Bone regeneration	11
Same pool of patients of another article	3
Number of patients < 10	1
Nonhuman study	9
Review	1
Language	4

**(a):** 

Author	Year	Study type	Study type 2	Follow-up mean (range)	Surgical intervention (surgical guide)	Implant system	Implant type	Abutments
Abbas H. et al.	2017	Prospective	Case series	4 years (nr)	Flapless (nr)	Implasa Höchests	Two pieces	Ball attachments
Aunmeungtong W. et al.	2016	Prospective	rct	1 year (nr)	Flapless (lab template)	pw plus	Two pieces	Equator
El-Sheikh AM et al.	2012	Prospective	Case series	2 years (nr)	Flap (nr)	Straumann	Two pieces	Locator abutment
Jawad S. et al.	2017	Prospective	rct	6 months (nr)	Flap (nr)	Astra	Two pieces	Ball attachments
Ma et al.	2016	Prospective	rct	7.6 years (1-10 years)	Flap (nr)	Nobel/southern	Two pieces	Ball attachments/u shape bar
Marcello-Machado et al.	2018	Prospective	Cohort	1 year (1 year)	Flap (nr)	Neodent	Two pieces	Equator
Morneburg	2008	Prospective	Cohort	6 years (1-9 years)	Flapless (lab template)	Microplant, Komet Brasseler Group	Two pieces	Magnetic/o-ring
Müller F. et al.	2015	Prospective	rct	5 years (3-6 years)	nr	Straumann	Two pieces	Locator abutment
Zweers et al.	2013	Retrospective	Cohort	3 years (nr)	Flap (lab template)	Straumann	Two pieces	Ball/locator

**(b):** 

Patient age—mean (restored arch)	Initial *N* of patients (*n* at the end of follow-up)	Initial *N* of rehabilitations (*n* of rehabilitations at the end of follow-up)	Initial *N* of implants (*n* of implants at the end of follow-up)	Implant diameter (implant length)	Loading	Prosthesis type
53.5 (mandible)	12 (12)	12 (12)	24 (24)	3.2 mm (11.5 mm)	Immediate <7 days and conventional	12 ovd on 2 implants
67.92 (mandible)	40 (40)	40 (40)	120 (120)	3 mm (12 mm)	Immediate <7 days	20 ovd on 2 implants; 20 ov on 4 implants
60.4 (mandible)	20 (20)	20 (20)	50 (50)	3.3 mm (10/12/14 mm)	Early 1-3 months	10 ovd on 2 implants/10 ov on 3 implants
68.5 (mandible)	24 (22)	24 (24)	48 (44)	3 mm (11 mm)	Early 1-3 months	ovd su 2 implants
64 (maxilla and mandible)	39 (23)	39 (23)	117 (69)	3.3 mm/3.25 mm (10/11.5/13/15 mm)	Conventional >3 months	ovd on 2 implants (mandible); old on 3 implants with bar or ball att. (maxilla)
67.5 (mandible)	30 (30)	30 (20)	60 (50)	2.9 mm (10 mm)	3 months	ovd on 2 implants
69 (mandible)	67 (61)	67 (61)	134 (122)	2.5 mm (09/12/15 mm)	Conventional >3 months	ovd on 2 implants
72 ± 8 (mandible)	91 (47)	91 (47)	182 (94)	3.3 mm (8/10/12/14 mm)	Early 1-3 months	ovd on 2 implants
69 (mandible)	75 (75)	75 (75)	150 (150)	3.3 mm (8/10/12/14 mm)	Early 1-3 months	ovd on 2 implants

**Table 5:** Risk of bias.

Author	Design	Random sequence generation	Allocation concealment	Blinding of participants and personnel	Blinding of outcome assessment	Incomplete outcome data	Selective reporting	Other bias
Abbas H. et al.	Prospective Case series	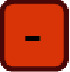	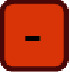	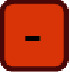	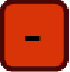	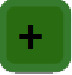	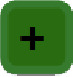	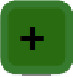
Aunmeungtong W. et al.	Prospective RCT	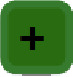	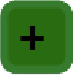	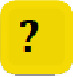	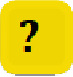	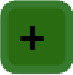	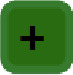	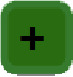
El-Sheikh AM et al.	Prospective	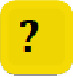	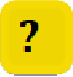	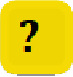	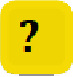	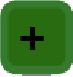	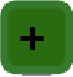	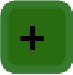
Jawad S. et al.	Prospective RCT	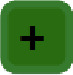	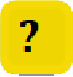	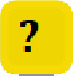	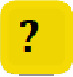	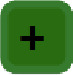	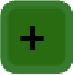	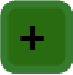
Ma et al. a-b	Prospective RCT	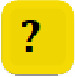	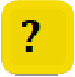	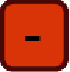	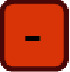	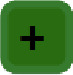	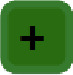	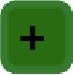
Marcello-Machado et al.	Prospective	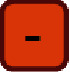	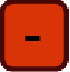	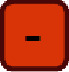	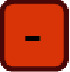	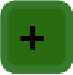	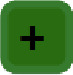	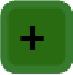
Morneburg	Retrospective Cohort	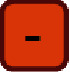	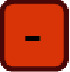	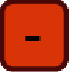	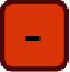	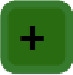	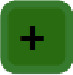	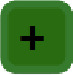
Müller F. et al.	Prospective RCT	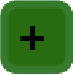	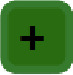	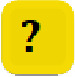	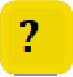	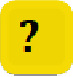	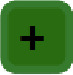	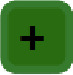
Zweers et al.	Retrospective Cohort	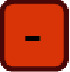	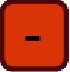	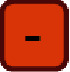	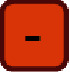	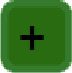	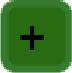	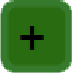

**Table 6:** Estimated implant survival rate and MBL at 5 years for the included studies.

Author	Implant survival rate reported	Estimated survival rate at 5 years (%)	MBL reported	Estimated mean MBL at 5 years	Prosthesis survival rate
Abbas H. et al.	95.8%	94.8%	1.25 mm	1.56 mm	NR
Aunmeungtong W. et al.	100%	100%	0.56 mm	2.82 mm	NR
El-Sheikh AM et al.	100%	100%	0.8 mm	2 mm	100%
Jawad S. et al.	100%	100%	NR	NR	100%
Ma et al.	84.7%	89.88%	2.45 mm	1.61 mm	NR
Marcello-Machado et al.	83.3%	81.7%	0 mm	0 mm	Nr
Morneburg	95.5%	96.27%	1.1 mm	0.92 mm	NR
Müller F. et al.	98.35%	98.35%	0.6 mm	0.6 mm	NR
Zweers et al.	100%	100%	0.48 mm	0.8 mm	100%
		Estimated cumulative survival rate at 5 years (%) Weighted mean 96.75%, median 98.35%, 95% CI: 90.87%, 100%		Estimated cumulative MBL at 5 years (%) Weighted mean 1.40 mm, median 1.24 mm, 95% CI: 0.55 mm, 2.03 mm	

**Table 7:** Life table analysis.

Interval (months)	Implants at risk	Failed implants	Dropouts/lost to follow-up	Implant survival rate	Cumulative survival rate
0–6	885	17	19	98.08%	98.08%
6–12	849	3	182	99.65%	97.73%
12–24	664	1	140	99.85%	97.59%
24–36	523	0	156	100.00%	97.59%
36–48	367	1	87	99.73%	97.32%
48–60	279	1	99	99.64%	96.97%
60–72	179	1	10–	99.44%	96.43%
72–84	168	1	0	99.40%	95.85%
84–96	167	0	6	100.00%	95.85%
96–108	161	0	125	100.00%	95.85%
108–120	36	0	36	100.00%	95.85%
